# Variety in the Daily Food

**Published:** 1883-12

**Authors:** 


### VARIETY IN THE DAILY FOOD.

Though good wheat, or good beef, or good milk, may each furnish a perfect food, or contain all of the elements needed to support life, it is not best to depend upon any one article of food alone, except in the case of nursing babes, and then the mother should have a suitable variety. To obtain a variety some housekeepers only go a round of different kinds of pie and cake, all equally bad, perhaps; thinking that if the pantry is well supplied with these things little other cooking is necessary. It is a great mistake. Cake and pie do not supply much actual food, and the good material that is used in them is put into such shape that the stomach is wearied and worn out by the effort to digest them. This accounts for much of the tiredness complained of by women and girls. They are half starved because their food is poor. The use of much poor trash called "dainties" (I don't abuse these things because I dislike them; I have a "sweet tooth," and know my own weakness well enough to understand the weakness of others), spoils the appetite for substantial food. The stomach is feeble for lack of good material in the blood (made constantly of our food and air) to repair its waste, and it takes food unwillingly because it is tired with overwork—overwork upon the concentrated conglomerations of rich cake and pastry. An error easily fallen into in such a case is to give up one thing after another because it "hurts" us, until the stomach becomes so weak it can hardly bear anything. It is slow starvation. We must not only "cease to do evil," but must also "learn to do well"—not only give up unwholesome food, but eat plenty of that which is wholesome.

The proper variety is one made up of fruits, vegetables, grain and and animal food, the latter consisting of healthy meat, eggs or milk in its various forms. With palatable graham or oatmeal prepartions, especially where milk is freely used, meat is seldom craved or found to be necessary to high health and strength, but when starch, sugar and fat preponderate, as in the common fare of white bread and butter, potatoes, cake, pie and a little sauce—beef (especially steak) often seems an absolute necessity to one who has to put forth strength. Coffee cannot possibly supply its place. It does not give strength, but only stimulates it, or calls it out, making one feel strong while under its influence. Nourishing foods really strengthen us.

You would hardly believe, until you try it, how heartily a plain and nourishing variety of food is enjoyed by those who live with reasonable simplicity. It is easier in every way. All feel much better and more good natured, with no unreasonable cravings for confectionary, pickles, or stimulants. It lightens the care of children wonderfully. It makes the cooking more simple and easy, and, last but not least, it saves the doctor's bills.

